# Clinical and imaging characteristics in patients undergoing surgery for lumbar epidural lipomatosis

**DOI:** 10.1186/s12891-018-1988-8

**Published:** 2018-03-01

**Authors:** Taketoshi Yasuda, Kayo Suzuki, Yoshiharu Kawaguchi, Shoji Seki, Hiroto Makino, Kenta Watanabe, Takeshi Hori, Tohru Yamagami, Masahiko Kanamori, Tomoatsu Kimura

**Affiliations:** 1 Department of Orthopaedic Surgery, Faculty of Medicine, University of Toyama, 2630 Sugitani, Toyama, Toyama 930-0194 Japan; 2 Department of Orthopaedic Surgery, Nippon Koukan Hospital, 1-2-1 Kokandori, Kawasaki, Kanagawa 210-0852 Japan; 3 Department of Orthopaedic Surgery, Itoigawa General Hospital, 457, Takegahana, Itoigawa, Niigata, 941-8502 Japan; 4 Department of Human Science 1, University of Toyama, 2630 Sugitani, Toyama, Toyama 930-0194 Japan

**Keywords:** Lumbar epidural lipomatosis, Clinical feature, Japanese Orthopaedic Association score, Laminectomy, Epidural space pressure, Computed tomography, Magnetic resonance imaging, Saucerization, Adipose tissue, Body mass index

## Abstract

**Background:**

Lumbar epidural lipomatosis (LEL) is characterized by abnormal accumulation of unencapsulated adipose tissue in the spinal epidural space. Such accumulation compresses the dural sac and nerve roots, and results in various neurological findings. However, the pathophysiology of LEL remains unclear. This study examined the associations between imaging and clinical findings in detail, and investigated the mechanisms underlying symptom onset by measuring intraoperative epidural pressures in LEL.

**Methods:**

Sixteen patients (all men; mean age, 68.8 years) were enrolled between 2011 and 2015. Mean body mass index was 26.5 kg/m^2^. Four cases were steroid-induced, and the remaining 12 cases were idiopathic. All patients presented with neurological deficits in the lower extremities. Cauda equina syndrome (CES) alone was seen in 8 patients, radiculopathy alone in 4, and both radiculopathy and CES (mixed CES) in 4. All patients subsequently underwent laminectomy with epidural lipomatosis resection and were followed-up for more than 1 year. We investigated the clinical course and imaging and measured epidural pressures during surgery.

**Results:**

Subjective symptoms improved within 1 week after surgery. Mean Japanese Orthopaedic Association (JOA) score was 15.2 ± 2.8 before surgery, improving to 25.4 ± 2.5 at 1 year after surgery. On magnetic resonance imaging, all lipomatosis lesions included the L4–5 level. On preoperative computed tomography, saucerization of the laminae was not observed in radiculopathy cases, whereas saucerization of the posterior vertebral body was observed in all radiculopathy or mixed CES cases. Intraoperative epidural pressures were significantly higher than preoperative subarachnoid pressures. The results suggest that high epidural pressure resulting from the proliferation of adipose tissue leads to saucerization of the lumbar spine and subsequent symptoms.

**Conclusions:**

Clinical courses were satisfactory after laminectomy. In LEL, epidural pressure increases and symptoms develop through the abnormal proliferation of adipose tissue. Higher epidural pressures induce saucerization of the laminae and/or posterior vertebral body. Furthermore, the direction of proliferative adipose tissue (i.e., site of saucerization) might be related to the types of neurological symptoms.

## Background

Spinal epidural lipomatosis (SEL) is a rare condition that was first described by Lee et al. in 1975 [1]. SEL is defined as the abnormal accumulation of unencapsulated adipose tissue in the spinal epidural space, and causes various neurological symptoms [2]. Secondary SEL occurs in relation to exogenous steroid treatment [2, 3], endocrinopathies such as Cushing syndrome [4], and hypothyroidism [5]. Idiopathic SEL may occur without any history of steroid use or endocrine disorder [6, 7]. The condition predominantly affects males, and more than 75% of all reported patients are obese [8–10]. However, factors involved in the onset of SEL remain unclear.

Criteria for clinical symptoms and imaging characteristics of SEL have not been firmly established. The neurological symptoms resemble degenerative lumbar spinal stenosis (LSS). Patients with LSS have recently been reported to show increased epidural pressure at the level of stenosis [11]. However, no reports have provided values for epidural pressure in the spinal canal of SEL patients. Generally, adipose tissue is soft, and is unlikely to provide substantial compression on the dural sac or nerve root. We therefore hypothesized that abnormal proliferation of adipose tissue within the spinal canal increases the epidural pressure and leads to symptoms in SEL.

Diagnosis of SEL is mainly based on the results of magnetic resonance imaging (MRI), which is considered the most sensitive modality for assessing adipose tissue [12, 13]. In the majority of cases, excessive deposition of epidural fat (EF) showing signal hyperintensity on T1-weighted imaging and intermediate signal intensity on T2-weighted imaging is found in the spinal epidural space [12]. A characteristic “Y-sign” or square stellate dural sac on axial MRI is often seen in lumbar epidural lipomatosis (LEL) [14]. Myelography and postmyelography computed tomography (CT) have also been applied for diagnostic purposes, but are not as sensitive as MRI [14, 15]. Myelography and CT myelography thus do not allow clear differentiation between LSS and LEL. However, few reports have described detailed imaging findings from MRI and CT for a large number of cases. Furthermore, no reports have clarified the relationship between image findings and symptoms. The purpose of this study was to examine imaging characteristics and measure epidural pressures in patients with LEL undergoing decompressive surgery. This study suggests that high epidural pressure resulting from the proliferation of adipose tissue leads to saucerization of the lumbar spine and subsequent symptoms.

## Methods

Sixteen patients underwent decompressive surgery for LEL in our institution between 2011 and 2015. The indication for surgery was neurogenic symptoms that failed to respond to conservative treatment for more than 3 months. We diagnosed LEL of more than grade II by Borré’s definition at the axial plane parallel to the superior end plate of the S1 vertebral body on T1-weighted axial MRI [12]. Briefly, Grade II represents moderate overgrowth of EF, defined as having an EF/spinal anteroposterior diameter (SpiC) index more than 50% at the spinal level responsible for symptoms. In contrast, cases with EF/SpiC index less than 50% were excluded. MRI was assessed by 2 different spine surgeons (T.Y. and K.S.). Furthermore, we excluded subjects who were treated with reduction operation of spinal deformities such as degenerative scoliosis with a Cobb angle more than 10 degrees; lumbar kyphosis with a Cobb angle more than 10 degrees, and spondylolisthesis with a Meyerding classification grade higher than 1 [16]. Demographic data are shown in Table 1. Slight LSS was recognized in some cases (Cases 3, 10, 12, 15, and 16), but severe degenerative LSS, in which epidural adipose tissue was absent at the level of the intervertebral disc and the dural sac was compressed, were excluded. Cases in which a herniated disc was excised were also excluded. Mean age was 68.8 years (range, 47–77 years) at the time of surgery. Four patients had a history of steroid administration and 12 patients had no history of steroid administration or endocrinopathies. Mean body mass index (BMI) was 26.2 kg/m^2^ (range, 25.2–27.7 kg/m^2^). All patients presented with neurological deficits in the lower extremities with intermittent claudication, as seen in cauda equina syndrome (CES) and/or radiculopathy (Table 2). Neural symptoms were classified into 3 types according to a previous report [17]: radicular type, presenting as unilateral radicular pain; cauda equina type, showing symptoms with less dermatomal-specific neurogenic claudication; and mixed type, showing characteristics of both radicular and cauda equine types. All patients subsequently underwent trumpet laminectomy [18] with epidural lipomatosis resection. Patients were followed-up for more than 1 year. The present study was approved by the ethics committee at Toyama University Hospital. Patients provided written informed consent to participate in this analysis.

**Table 1:** Profiles of patients with lumbar epidural lipomatosis

Case	Age (years)	Medical history	History of steroid administration	BMI (kg/m^2^)
1	64–65	none	none	26.1
2	79–80	gout	none	26.2
3	77–78	none	none	27.1
4	69–70	none	none	26.5
5	70–71	prostatic hypertrophy	none	25.6
6	73–74	none	none	25.8
7	77–78	hypertension	none	26.6
8	69–70	none	none	26.9
9	47–48	none	none	27.7
10	73–74	none	none	25.2
11	69–70	none	none	25.8
12	61–62	none	none	25.8
13	70–71	sudden deafness	PSL 5 mg/day for 1 year	25.5
14	77–78	polymyalgia rheumatica	PSL 7.5 mg/day for 3 years	26.2
15	62–63	interstitial pneumonia	PSL 8 mg/day for 1 year	27.6
16	64–65	Behçet disease	PSL 8 mg/day for 12 years	25.5
Mean	68.8			26.2

All patients are men. *BMI* body mass index, *PSL* prednisolone

**Table 2:** Patients’ clinical data

Case	LEL levels	LEL grade	Duration of symptoms (months)	Level (s) of neural disorder	Type of neural disorder
1	L2–S1	3	8	L5	Cauda equina
2	L2–S1	3	48	L3	Cauda equina
3	L3–S1	3	5	L5, S1	Radicular
4	L3–S1	2	36	L4	Cauda equina
5	L3–S1	2	24	L5, S1	Radicular
6	L2–S1	3	10	L4	Cauda equina
7	L3–5	2	4	L5	Mixed
8	L4–S1	3	6	L4	Cauda equina
9	L2–5	3	6	L3	Radicular
10	L2–5	2	3	L4	Radicular
11	L3–5	2	15	L4	Mixed
12	L2–S1	2	10	L4	Cauda equina
13	L2–S1	3	9	L5	Mixed
14	L3–S1	3	5	L4	Cauda equina
15	L3–S1	2	3	L4	Cauda equina
16	L3–5	2	8	L4	Mixed

*LEL* lumbar epidural lipomatosis, *L* lumbar, *S* sacral

### Clinical evaluation

We investigated the duration of subjective symptoms, levels of neural disorder, and types of neural disorders before surgery. Furthermore, the relationship between the symptomatic level and the most severely stenotic level on MRI was analyzed.

Clinical courses were evaluated using the Japanese Orthopaedic Association (JOA) scores at 3 months, 6 months, and 1 year after surgery (Table 3) [19]. Briefly, subjective symptoms (9 points: low back pain, leg pain, and walking ability), clinical signs (6 points: sensory and motor disturbance and positive straight leg raise test results), and activities of daily living (ADL, 14 points) were evaluated. The ratio of recovery, which indicates the degree of normalization after surgery, was calculated using the following formula: (postoperative score − preoperative score) × 100 / [29 (full score) − preoperative score] [20].

**Table 3:** Scoring system for the treatment of low back disorders devised by the Japanese Orthopaedic Association

Item	Score		
Subjective symptoms			
Low back pain			
None	3		
Occasional mild pain	2		
Frequent mild or occasional severe pain	1		
Frequent severe pain	0		
Leg pain and/or numbness			
None	3		
Occasional mild leg pain and/or numbness	2		
Frequent mild or occasional severe leg pain and/or numbness	1		
Frequent severe leg pain and/or numbness	0		
Walking capacity			
Normal	3		
Able to walk > 500 m with leg pain and/or numbness	2		
Able to walk 100–500 m with leg pain and/or numbness	1		
Able to walk < 100–500 m with leg pain and/or numbness	0		
Clinical signs			
Straight leg raise test			
Normal	2		
30 degrees to 70 degrees	1		
< 30 degrees	0		
Motor function			
Normal	2		
Slight weakness (MMT: good)	1		
Severe weakness (MMT: poor)	0		
Sensory function			
Normal	2		
Slight disturbance	1		
Severe disturbance	0		
Bladder function			
Normal	0		
Mild dysuria	–3		
Severe dysuria	–6		
Restriction of activities of daily living	Impossible	Difficult	Easy
Turning in bed	0	1	2
Standing up	0	1	2
Washing face	0	1	2
Half-sitting posture	0	1	2
Sitting	0	1	2
Lifting	0	1	2
Running	0	1	2
Total for normal	29		

*MMT* manual muscle testing

### Imaging analysis

#### Magnetic resonance imaging

**Fig. 1:**
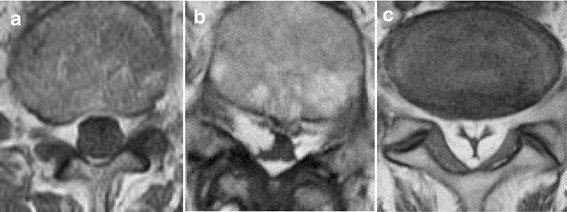
Classification of shape of dural sac on axial T1-weighted magnetic resonance imaging. **a** Circular type, **b** square stellate type, **c** “Y-sign”

In all 16 cases, MRI was performed within 1 month before surgery. In cases whose symptoms persisted more than 6 months, MRI was re-examined, but there was no major change. We evaluated the most severely stenotic level, LEL level and grade, and shape of the dural sac on the latest MRI. LEL grade was classified based on midsagittal T1-weighted MRI (MAGNETOM Avanto; Siemens, München, Germany) under a magnetostatic field intensity of 1.5 T [12]. Shape of the dural sac was divided into 3 types: circular; square stellate; or “Y-sign” at the level of each intervertebral disc and middle of the vertebral body in which LEL was present on T1-weighted axial MRI, according to a previous report (Fig. 1) [12].

#### Computed tomography

**Fig. 2:**
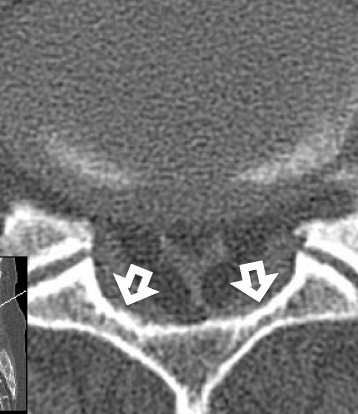
Saucerization of laminae on computed tomography. Saucerization of laminae is defined as a dome-formed spinal canal with thinness of the vertebral lamina (arrows)

**Fig. 3:**
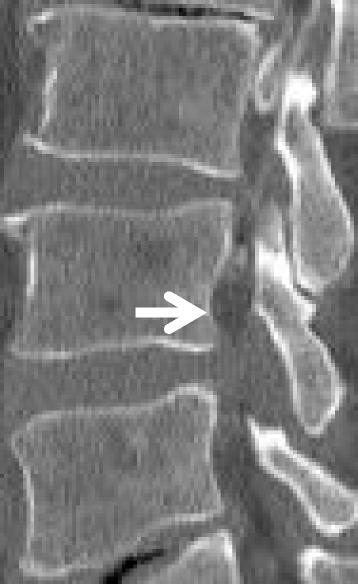
Saucerization of posterior vertebral body on computed tomography (CT). Saucerization of posterior vertebral body is defined findings of compression of more than 2 mm than the usual posterior body of a vertebra in the reconstructed sagittal CT view (arrow)

In 13 cases, myelography and CT myelography were performed before surgery within 1 week. We evaluated the presence of saucerization of the laminae or/and posterior vertebral body. Saucerization of the laminae was defined as a dome-formed spinal canal with thinness of the vertebral lamina at the level of each involved intervertebral disc on axial view (Fig. 2). Saucerization of the posterior vertebral body was defined as a finding of compression more than 2 mm compared to usual for the posterior vertebral body in the reconstructed sagittal view (Fig. 3). CT findings were assessed by two different spine surgeons (T.Y. and K.S.).

### Preoperative subarachnoid pressure and intraoperative epidural pressure

Preoperatively, subarachnoid pressures were measured at the L2 − 3 level during myelography. Measurement was performed with patients in the left decubitus position.

We measured intraoperative epidural pressure at L4–5, as previously described [11]. Briefly, surgery was performed with patients in the prone position, using a standard posterior midline approach. Prior to the actual surgical intervention, an arterial line (A-line) catheter was first calibrated in accordance with the instructions from the manufacturer, then inserted through an interlaminar window at the L4–5 level. Complete flavectomy had not been performed at this point to allow correct measurement of pressure. Because pressure values during positioning of the catheter fluctuate considerably due to mechanical loading at the tip of the catheter, measurements were taken after a time lag of 10 s after positioning of the catheter. Pressure values were recorded at half the height of the vertebral body. Measurements were performed 3 times, and the average of the 3 measurements was calculated. Patients provided written informed consent for intraoperative measurement of epidural pressure, which required an extension of the operating time by approximately 15 min. We compared intraoperative epidural pressures to preoperative subarachnoid pressures.

### Statistical analysis

Values are expressed as means ± standard deviation (SD). Significant differences between means were analyzed using the Mann-Whitney’s U test, and a *P* value less than 0.05 was considered statistically significant.

## Results

### Clinical evaluation

#### Preoperative symptoms

Mean duration of symptoms before surgery was 13.1 months (range, 3–48 months) (Table 2). Among the 16 patients, the level of neural disorder was L3 in 2 patients (13%), L4 in 9 patients (56%), and L5 and/or S1 in 5 patients (31%). Types of neural disorder were categorized as cauda equina type in 8 patients (50%), mixed type in 4 (25%), and radicular type in 4 (25%). In 12 patients (75%), the level of neural disorder corresponded to the most stenotic level.

#### Clinical course

In all patients, subjective symptoms improved within 1 week after surgery. Mean JOA scores were 15.2 ± 2.8 preoperatively, 21.3 ± 2.2 at 3 months after surgery, 24.6 ± 1.2 at 6 months, and 25.4 ± 2.5 at 1 year. The ratio of recovery at 1 year after surgery was 70.9 ± 5.2%. No recurrent and/or worsening cases were identified during follow-up.

### Imaging evaluation

#### Magnetic resonance imaging findings

LEL existed from L2-S1 in 5 patients (31%), L2–5 in 2 (13%), L3-S1 in 5 (31%), L3–5 in 3 (19%), and L4-S1 in 1 (6%) (Table 4). All lipomatosis lesions included the L4–5 level. LEL was grade II in 8 patients (50%) and grade III in 8 (50%). Shapes of the dural sac are shown in Table 4. A square stellate shape was seen at the L4 and L4–5 levels, and the “Y-sign” was seen only at the L5-S level.

**Table 4:** Magnetic resonance imaging (MRI) and computed tomography (CT) findings

Level	Number of total cases	Shape of the dural sac on MRI			Saucerization on CT	
		Circular	Square stellate	“Y-sign”	laminae	Posterior vertebral body
L2	7	6	1	0		0
L2–3	7	6	1	0	0	
L3	15	10	5	0		0
L3–4	15	9	6	0	3	
L4	16	5	10	0		6
L4–5	16	3	13	0	12	
L5	16	8	8	0		0
L5-S1	10	0	2	8	10	

*L* lumbar, *S* sacral

#### Computed tomography findings

Saucerization of the laminae was recognized in 14 of the 16 patients (88%), at L3–4 in 3 patients (20%), L4–5 in 12 (75%) and L5-S1 in 10 (63%), with some overlap of levels (Table 4). Saucerization of the posterior vertebral body was recognized in 6 patients (56%), at the L4 level in all cases. With regard to the relationship between subjective symptoms and CT findings, the 2 patients in whom saucerization of the laminae was not seen had radiculopathy (Cases 3 and 9). Furthermore, symptoms of patients with saucerization of the posterior vertebral body were radicular or mixed type (Cases 1, 3, 5, 7, 9, 10, and 11).

### Preoperative subarachnoid oressures and intraoperative epidural pressures

Mean preoperative subarachnoid pressure (standard value: 7–18 cmH_2_O) was 12.0 ± 2.8 cmH_2_O, and mean intraoperative epidural pressure (standard value: − 3 to 1 cmH_2_O) was 42.9 ± 14.8 cmH_2_O (Table 5). Intraoperative epidural pressures were significantly higher than preoperative subarachnoid pressures (Student’s *t* test, *P* < 0.001). In patients with lipomatosis alone, mean preoperative subarachnoid pressure was 11.3 ± 3.0 cmH_2_O and mean intraoperative epidural pressure was 46.1 ± 15.6 cmH_2_O. In patients with both slight LSS and LEL, mean preoperative subarachnoid pressure was 13.5 ± 1.7 cmH_2_O and mean intraoperative epidural pressure was 35.0 ± 10.0 cmH_2_O. No significant difference was seen between patients with LEL alone and patients with both slight LSS and LEL.

**Table 5:** Preoperative subarachnoid pressures and intraoperative epidural pressures

Case	Subarachnoid pressure (cm H_2_O)	Epidural pressure (cm H_2_O)
1	7	57
2	7	65
3	15	30
4	11	38
5	NA	NA
6	13	34
7	NA	23
8	16	38
9	13	43
10	NA	NA
11	12	62
12	12	48
13	13	68
14	10	33
15	12	25
16	15	37
Mean ± SD	12.0 ± 2.8	42.9 ± 14.8

*NA* not available, *SD* standard deviation

### Illustrative case (case 15)

**Fig. 4:**
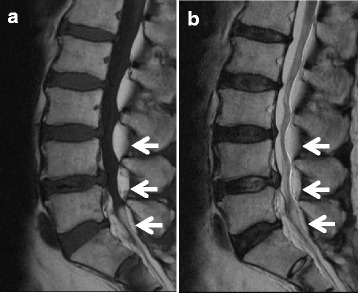
Lumbar sagittal magnetic resonance imaging. **a** T1-weighted sagittal image, **b** T2-weighted sagittal image. Arrows indicate lumbar epidural fat

**Fig. 5:**
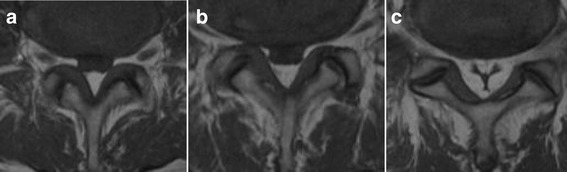
Lumbar axial magnetic resonance imaging. The adipose tissue compresses the thecal sac at L3–S1 levels, circumferentially. **a** L3-4 level, **b** L4-5 level, **c** L5-S1 level

**Fig. 6:**
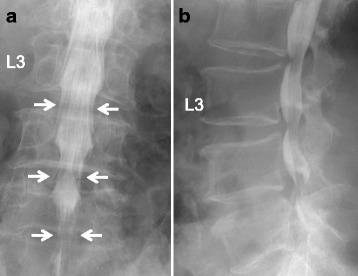
Myelogram of the lumbar spine. Myelogram shows tapering of the dural sac at the L3 − S1 levels (arrows). **a** Anterior‑posterior view, **b** lateral view

**Fig. 7:**
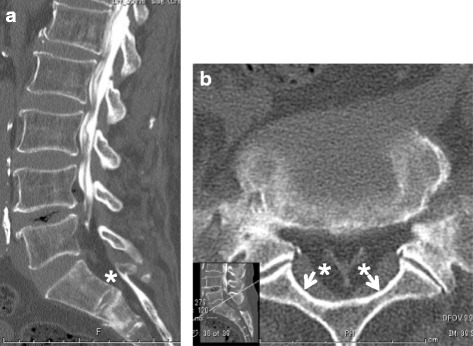
Computed tomography myelogram. **a** Reconstructed sagittal view, **b** axial view. Saucerization of the laminae (arrows) with a homogeneous hypodense epidural mass (asterisks) is shown at the L5-S1 level

**Fig. 8:**
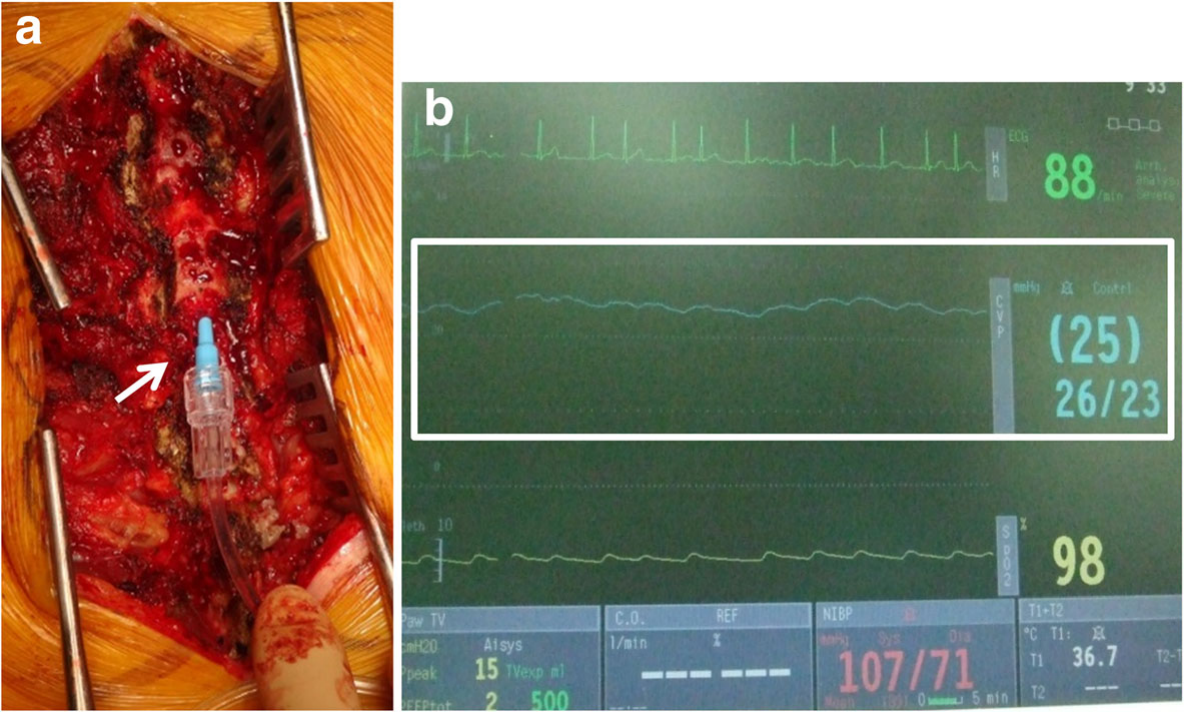
Intraoperative findings. **a** Arterial line catheter (arrow) is introduced into the epidural space above the dural sac at an L4–5 interlaminar window. **b** The epidural pressure shows the fluctuation depending on blood pressure, and it is measured with an average of 25 mm H_2_O (square)

A 62-year-old man was admitted to our hospital with bilateral leg pain. He had a 3-month history of intermittent low back pain accompanied by asymmetrical, slowly progressive bilateral leg pain. The neural disorder was diagnosed as L4 cauda equina type and the preoperative JOA score was 14. The patient was slightly overweight, with a BMI of 27.6 kg/m^2^. He had been receiving oral prednisolone at 8 mg/day for interstitial pneumonia for 8 years. Lumbar radiography showed slight spondylosis. Lumbar MRI revealed moderate disc bulging at the L3-L5 levels (Fig. 4). A high-intensity mass lesion on T1- and T2-weighted imaging consistent with LEL was seen between L3 and S1. The mass extended both anteriorly and posteriorly and markedly compressed the dural sac with square stellate deformation of the dural sac at the L3–4 and L4–5 disc levels (Fig. 5a, b). The typical “Y-sign” [18] was seen at the L5-S1 level (Fig. 5c), and MRI was categorized as grade 3. Myelography showed tapering of the dural sac at the L3 − S1 levels (Fig. 6). CT myelography showed saucerization of the laminae at the L5-S1 level (Fig. 7). Preoperative subarachnoid pressure was 15 cm H_2_O. Laminectomies with LEL resection were performed at L3 − S1. Proliferation of EF was recognized. Intraoperatively, epidural pressure was measured at the L4–5 level (Fig. 8a), and was 25 cmH_2_O (Fig. 8b). Symptoms improved immediately after surgery, and the patient was able to walk normally thereafter. At 1 year after surgery, JOA score was 25 and the ratio of recovery was 73%. The histopathological findings consisted of proliferation of mature adipocytes, and LEL was diagnosed.

## Discussion

In this study, clinical courses of patients with LEL were satisfactory after laminectomy. In LEL, epidural pressure increases and symptoms develop through the abnormal proliferation of adipose tissue. The higher epidural pressure induces saucerization of the laminae and/or posterior vertebral body. Furthermore, the direction of proliferative adipose tissue (i.e., site of saucerization) might be related to the types of neurological symptoms.

SEL is a rare disorder characterized by overgrowth of adipose tissue in the extradural space causing compression of the neural elements [2]. This condition is frequently associated with the administration of exogenous steroids or elevation of endogenous steroids [2, 3]. Some cases have emerged without evidence of any clear predisposing factors [6–8]. Most patients with idiopathic SEL are obese [8–10], and more men than women are affected [14]. Steroid-induced SEL has been found to involve the thoracic spine, while idiopathic SEL has been found to predominantly involve the lumbar spine, i.e., LEL [2]. LEL is found in 39–42% of patients with SEL, and L4–5 is the most commonly involved level [21–23]. Few reports have described cervical SEL [3], and the pathogenesis of SEL has not yet been clarified. Frogel et al. classified SEL into 4 categories: 1) “exogenous steroid use” group, 55.3%; 2) “obesity only” group, 24.5%; 3) “endocrinopathy or endogenous steroid” group, 3.2%; and 4) “non-obese idiopathic” group, approximately 17% [2]. Four of our patients were consistent with the “exogenous steroid use” group and 12 were in the “obesity only” group. Furthermore, mean BMI was 26.8 kg/m^2^ (range, 25.5–27.7 kg/m^2^), and all 16 patients were overweight.

In patients with LEL, neurological symptoms and signs are similar to degenerative LSS: nonspecific low back pain; radicular pain; numbness and dysesthesias; neurogenic claudication; and CES [12]. In most reported cases, including ours, symptoms show a gradual onset [24, 25] and LEL is sometimes associated with other substantial pathological findings, such as disc herniation and lumbar deformity. The diagnosis of LEL is thus often delayed or missed [26]. In our cases, mean duration of diagnosis was delayed, at 13.1 months.

Imaging criteria for LEL have not been firmly established. CT and myelography do not provide specific findings for LEL, but may be useful as diagnostic support tools. On CT, LEL presents as a homogeneous, hypodense, epidural mass [27]. Lumbar myelography shows narrowing of the dural sac, which in the absence of bony stenosis on CT myelography indicates an epidural mass [28]. MRI is the most useful diagnostic imaging tool. Borré et al. reviewed the relationship between axial T1-weighted MRI at the L5-S1 level and onset of symptoms in 2258 LEL cases [12]. With regard to the relationship between EF/SpiC index and onset, symptomatic cases were not seen with Grade I (EF/SpiC, 41–50%), while symptoms were seen in 14% of Grade II cases (EF/SpiC, 51–74%) and all Grade III cases (EF/SpiC, ≥75%). Kuhn et al. reported the Y-sign as a specific characteristic of LEL on axial T1-weighted MRI [14]. In our cases, the Y-sign was recognized in only 7 patients (44%). Usually, MRI of the spine is performed with basic sequences including T1- and T2-weighted sagittal and axial imaging. Because the abnormal accumulation of adipose tissue in the spinal epidural space is shown in LEL, specific sequences such as fat-suppressed T2-weighted imaging might be helpful for differentiation from other epidural lesions [29]. Moreover, diffusion-weighted imaging and MR neurography might be useful to detect more spinal pathologies and evaluate nerve roots and courses. Further study is necessary on MRI.

Other characteristics of imaging for improved diagnosis of LEL are necessary. To the best of our knowledge, no reports have described CT findings of LEL. The present study clarified saucerization of the laminae and posterior vertebral body on CT for the first time. In addition, patients in whom CT did not show saucerization of laminae had radicular type, while patients showing saucerization of the posterior vertebral body showed radicular or mixed type. These findings might be useful for the diagnosis and symptoms of LEL.

To the best of our knowledge, no published data on epidural pressures in the spinal canal of LEL patients are available. Barz et al. reported a median epidural pressure of 30.7 cmH_2_O at the level of stenosis in LSS [11]. In our study, mean epidural pressure was 42.9 cmH_2_O at the L4–5 level in LEL. Epidural pressure was higher in LEL than in LSS. Furthermore, epidural pressure was higher than subarachnoid space pressure in LEL. These findings suggest that the increase in unencapsulated epidural adipose tissue in the spinal canal, which is a closed space, may increase the epidural pressure, and may subsequently lead to the onset of symptoms. The increase in pressure leads to saucerization of the laminae and/or posterior vertebral body. Increased pressure toward the dorsal side (central side) is speculated to lead to saucerization of the laminae, while pressure toward the foraminal side leads to saucerization of the posterior vertebral body. In this study, the site of saucerization was related to subjective symptoms before operation. Specifically, LEL with saucerization of the laminae led to CES, and LEL with saucerization of the posterior vertebral body led to radiculopathy.

Two treatment options for SEL have been recommended: conservative therapy and surgical intervention [24]. No clinical trials have compared outcomes of both modalities, because of the limited number of cases. Conservative treatment with controlled weight loss for obesity and/or reduction of steroids where possible has been reported [10, 27]. Conservative treatment should be undertaken before surgical intervention. Decompressive surgery with fat resection can be considered when conservative treatment is unsuccessful or when a patient exhibits acute neurological deterioration [25, 26]. Improvement and resolution of the neurological symptoms after surgery have been reported [26]. This study represents one of the largest series of patients with operatively treated symptomatic LEL. Al-Omari et al. reported that no difference between LEL and LSS in terms of operation results such as surgical complications, bleeding during surgery and outcomes [26]. In this study, surgical results were satisfactory during follow-up.

The present study has several limitations. The first limitation is to include LEL with sight LSS. However, all cases satisfied previously reported diagnostic methods [12, 14]. Furthermore, diagnostic criteria for LEL have yet to be defined. The second limitation was the lack of a control group. Third, the cohort was relatively small and future studies should examine a larger sample.

## Conclusions

In summary, LEL is defined as the abnormal accumulation of unencapsulated adipose tissue in the spinal epidural space, potentially causing various neurological symptoms. Due to the abnormal proliferation of adipose tissue, epidural pressure increases and symptoms can develop. Furthermore, the higher epidural pressure results in saucerization of the laminae and/or posterior vertebral body. The direction of proliferation (i.e., site of saucerization) might cause specific symptoms. Future additional studies of larger populations are necessary.

## Abbreviations

ADL: Activities of daily living
A-line: Arterial line
BMD: Body mass index
CES: Cauda equina syndrome
CT: Computed tomography
EF: Epidural fat
i.e.: Id est.
JOA: Japanese Orthopaedic Association
LEL: Lumbar epidural lipomatosis
LSS: Lumbar spinal stenosis
MRI: Magnetic resonance imaging
SD: Standard deviation
SEL: Spinal epidural lipomatosis
